# Challenging the thorium-immobility paradigm

**DOI:** 10.1038/s41598-019-53571-x

**Published:** 2019-11-19

**Authors:** Haylea Nisbet, Artas A. Migdisov, Anthony E. Williams-Jones, Hongwu Xu, Vincent J. van Hinsberg, Robert Roback

**Affiliations:** 1 Earth and Environmental Sciences Division, Los Alamos National Laboratory, Los Alamos, NM 87545 USA; 2 Department of Earth and Planetary Sciences, McGill University, 3450 University Street, Montreal, QC H3A 0E8 Canada

**Keywords:** Geochemistry, Economic geology

## Abstract

Thorium is the most abundant actinide in the Earth’s crust and has universally been considered one of the most immobile elements in natural aqueous systems. This view, however, is based almost exclusively on solubility data obtained at low temperature and their theoretical extrapolation to elevated temperature. The occurrence of hydrothermal deposits with high concentrations of Th challenges the Th immobility paradigm and strongly suggests that Th may be mobilized by some aqueous fluids. Here, we demonstrate experimentally that Th, indeed, is highly mobile at temperatures between 175 and 250 °C in sulfate-bearing aqueous fluids due to the formation of the highly stable Th(SO_4_)_2_ aqueous complex. The results of this study indicate that current models grossly underestimate the mobility of Th in hydrothermal fluids, and thus the behavior of Th in ore-forming systems and the nuclear fuel cycle needs to be re-evaluated.

## Introduction

Thorium is primarily concentrated in highly evolved geologic systems, owing to its high charge/radius ratio, which inhibits incorporation into common rock-forming minerals. A consequence is the association of Th with other incompatible elements, particularly the Rare Earth Elements (REE). In the past decade, a mounting interest in the REE (elements that have become critical commodities for a wide range of advanced technologies^1^), has prompted researchers and the mining industry to address the behavior of Th. This element, and to a lesser extent U, is known to incorporate into REE ore minerals and, in some cases, reach concentrations in these minerals exceeding 20 wt. % ThO_2_ (e.g. monazite). Owing to its intrinsic radioactivity, Th can dictate the economic viability of an ore deposit, making some impossible to mine. Therefore, the ability to predict the behavior of Th in natural systems and identify the conditions at which Th-depleted REE ores can form is crucial for the discovery of new, economic deposits of these highly sought-after elements. As many REE ore deposits are known to have formed by hydrothermal fluids^2–4^, knowledge of Th solubility and mobility in these fluids is essential.

Evidence of hydrothermal deposits with high concentrations of Th^5–7^ and rocks to which Th has been added or removed hydrothermally^8^ argue convincingly that this actinide can be readily mobilized in hot, aqueous solutions. However, existing models, which rely on extrapolations of low-temperature solubility data^9–14^ to high temperature, are unable to explain the observed mobility of Th in either natural or man-made settings. This contradiction arises because information on the behavior of Th in hydrothermal fluids is effectively non-existent, because of a lack of data on aqueous Th speciation at elevated temperature and pressure. This information gap has led us to recently begin investigating the solubility and speciation of Th in aqueous solutions at elevated temperature^15,16^.

In addition to ore-forming hydrothermal systems, understanding the behavior of Th is relevant for nuclear energy and other nuclear applications^17^. While underappreciated in the past, Th is now considered to be one of the most promising alternative fuels by the nuclear industry, which is undergoing a major transformation as it seeks to become safer, cleaner and more sustainable. Not only is Th four times more abundant in nature than uranium, it also offers a cleaner energy source that produces significantly less waste and is highly resistant to nuclear proliferation^18^. Although the idea of Th nuclear reactors is not new and, indeed, has been discussed since the introduction of nuclear energy, the push to develop Th nuclear technology has been particularly strong in the past few decades^18^, requiring that we greatly expand our knowledge of Th chemistry at relevant conditions.

The experimental study reported in this contribution presents essential high temperature (175–250 °C) thermodynamic data on the stability of Th-sulfate species in sulfate-bearing solutions. This speciation was selected for study because it is known that sulfate has a high affinity for Th at ambient temperature^19^ and because sulfate is known to occur in high concentrations in some hydrothermal fluids^20,21^.

## Results

**Figure 1:**
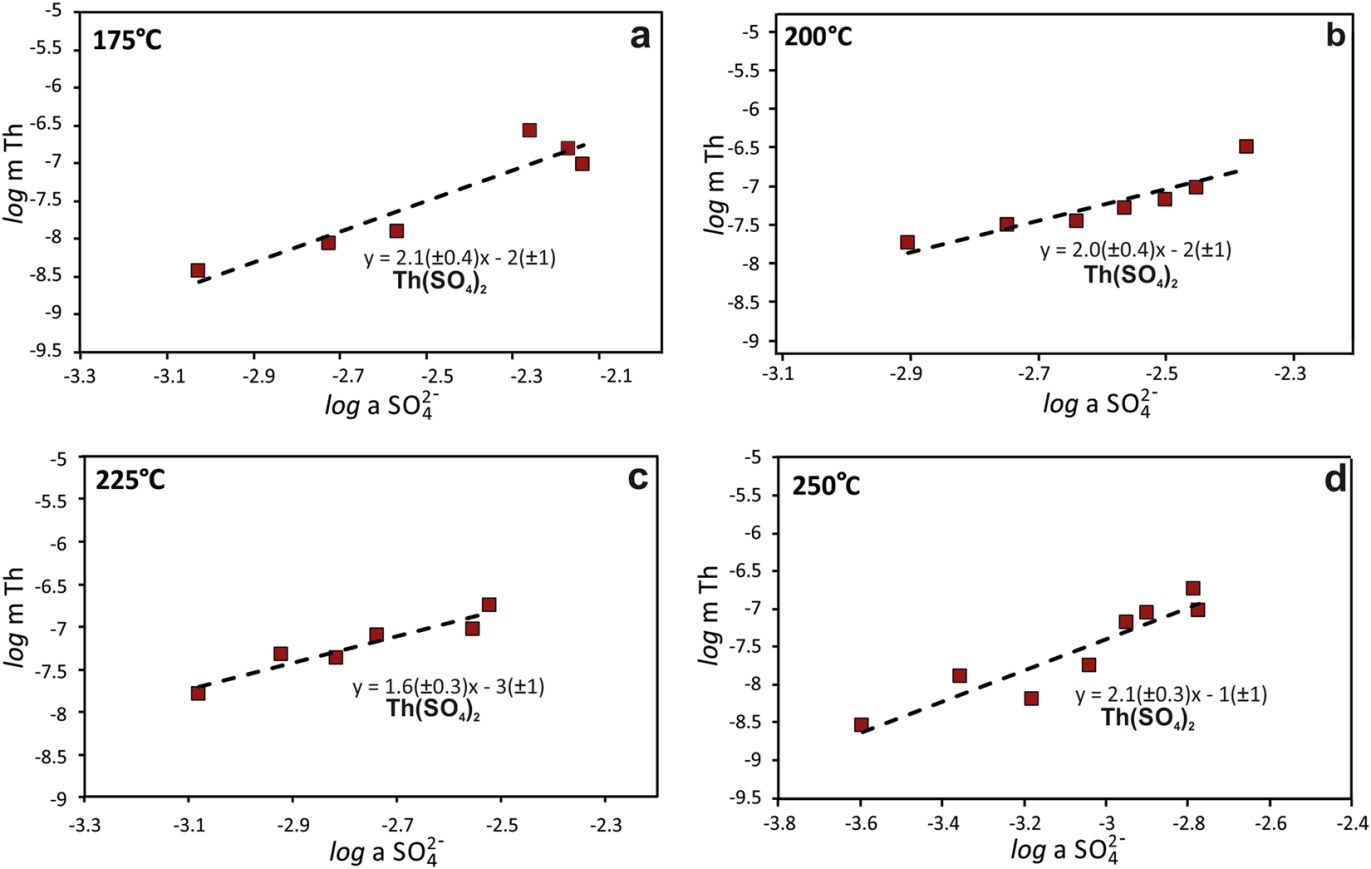
The solubility of ThO_2_ as a function of sulfate activity. The concentration of Th plotted as a function of the activity of sulfate determined at (**a**) 175 °C, (**b)** 200 °C, (**c)** 225 °C, and (**d)** 250 °C. Each data point represents the concentration of dissolved Th measured in an individual autoclave. The trend lines represent the fit of the data and have a slope of ~2. The error (SD) associated with the individual points is smaller than the markers.

In this study, we determined the solubility of Th dioxide (ThO_2_) in aqueous solutions as a function of sulfate concentration from 0.05 to 0.5 m Na_2_SO_4_ at temperatures between 175 and 250 °C and at the pressure of vapor-saturated water. Our results indicate that the amount of Th that can be dissolved in sulfate-bearing solutions is remarkably high, and, moreover, increases with the activity of sulfate at each temperature investigated (Fig. 1). This dependence provides compelling evidence that Th forms very stable complexes with sulfate. For each isotherm, the concentration of Th increases with respect to the activity of sulfate in a ratio of 2:1. Based on this ratio, the species controlling the ThO_2_ solubility in our experiments is interpreted to be Th(SO_4_)_2_, which formed via the reaction:

1 $${{ThO}}_{2}(s)+2{{SO}}_{4}^{2-}+4{{H}}^{+}\leftrightarrow {Th}{({{SO}}_{4})}_{2}+2{{H}}_{2}{O}$$

The experimental data (Supplementary Table S1) plotted in Fig. 1 were used to calculate equilibrium constants (log K) for the above reaction at each temperature. Formation constants (log β_2_) relating the predominant species in solution, Th(SO_4_)_2_, to the activity of Th^4+^ and SO_4_^2−^ were calculated using the following reaction (Table 1):

2 $${{Th}}^{4+}+2{{SO}}_{4}^{2-}\leftrightarrow {Th}{({{SO}}_{4})}_{2}$$

3 $${\log {\beta }}_{2}=\,\log \,{a}_{{Th}{({{SO}}_{4})}_{2}}-\,\log \,{a}_{{{Th}}^{4+}}-2\,\log \,{a}_{{{SO}}_{4}^{2-}}$$

**Table 1:** Calculated logarithm of equilibrium constants (log K) and formation constants (log β).

Reaction		175 °C	200 °C	225 °C	250 °C
$Th{O}_{2}+2S{O}_{4}^{2-}+4{H}^{+}\leftrightarrow Th{(S{O}_{4})}_{2}+2{H}_{2}O$	log K	12.79	14.45	15.67	15.86
$T{h}^{4+}+2S{O}_{4}^{2-}\leftrightarrow Th{(S{O}_{4})}_{2}$	log *β*_2_	17.48	19.83	21.70	22.50
	Uncertainty	± 0.47	± 0.53	± 0.25	± 0.52

Equilibrium constants calculated according to the associated reaction for each experimental temperature investigated, along with the derived uncertainty.

For further information on the calculation of the formation constants, readers are referred to the Supplementary Information file.

**Figure 2:**
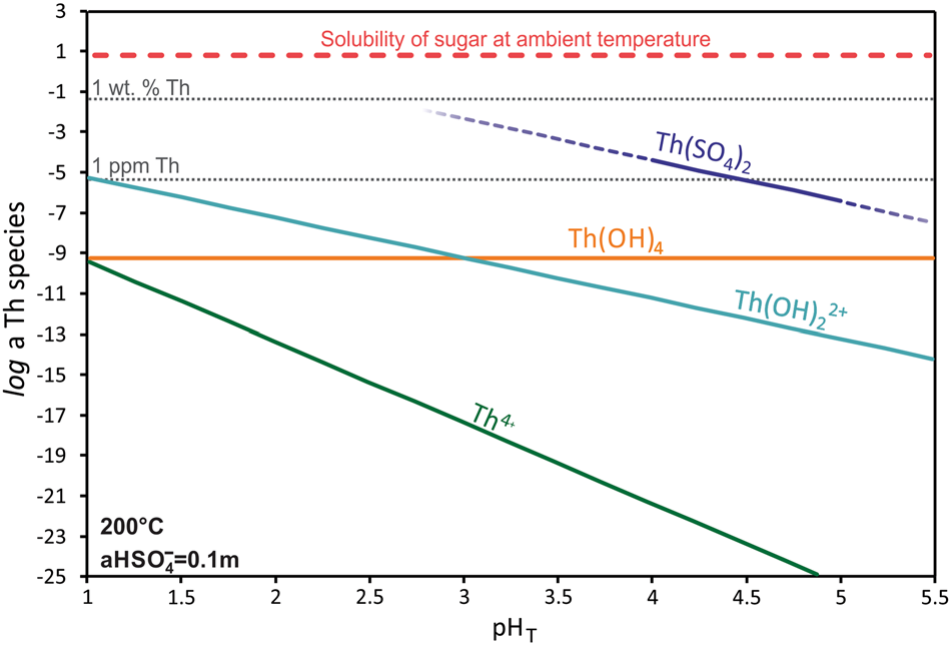
A model of aqueous Th speciation. The modeled stability of aqueous Th species as a function of pH in a 1 L solution with an activity of HSO_4_^−^ fixed at 0.1 m (concentrations after formation of sulfate complexes of Th) at 200 °C. The solid line for Th(SO_4_)_2_ corresponds to the experimentally investigated range of pH, whereas the dashed lines indicate estimates of Th concentration beyond the measured range.

The results indicate that under the conditions of our experiments, Th-sulfate complexes completely predominate over Th-hydroxyl complexes, rendering ThO_2_ extremely soluble. It should be noted that the contribution of Th-Cl complexes to the solubility of Th has been ignored in our data treatment as we have shown previously^15^ that Th-Cl complexes have very low stability. It is well-known that with decreasing pH, the total concentration of dissolved Th increases as a result of the higher concentration of Th^4+^ ^19^, and that this leads to a higher concentration of Th complexes (e.g. Reaction 2). Indeed, based on our stability constants for Th(SO_4_)_2_ at 200 °C and a pH of 2, 1 L of solution with the activity of HSO_4_^−^ fixed at 0.1 m is predicted to dissolve more than 2 kg of ThO_2_ (Fig. 2). This exceptionally high solubility is comparable to that of sugar (2039 g/L at 20 °C)^22^ in water at room temperature! Although such high Th concentrations are highly unlikely in natural systems (they would require that a stoichiometric equivalent amount of sulfate be present in the solution), these simplistic calculations illustrate the conditions that lead to the exceptionally high solubility of ThO_2_, even at temperatures as low as 200 °C. It should be noted that in addition to Th and SO_4_^2−^, our system also contains Si and Ti. However, the observed trends in the experimental data (Fig. 1) do not suggest any significant contribution of these elements to the solubility of Th.

Our results suggest that previous studies have grossly underestimated the mobility of Th in natural systems, requiring that existing hypotheses for the behavior of Th in nature be re-evaluated. It also requires that the implementation of nuclear protocols that may lead to the interaction of Th and sulfate be carefully assessed.

## Discussion

Given that the feasibility of exploiting a REE deposit economically is largely governed by the concentration of Th, identifying conditions under which Th-depleted REE deposits form is a pre-requisite for successful exploration. Modern exploration is based on genetic models that predict geochemical controls on metal enrichment. In the case of REE exploration, an additional requirement is that the models also predict the geochemical controls on Th depletion. Existing models for REE ore genesis have been unable to discriminate between Th-rich and Th-depleted deposits because of the false assumption that Th is effectively immobile in hydrothermal fluids^16^.

**Figure 3:**
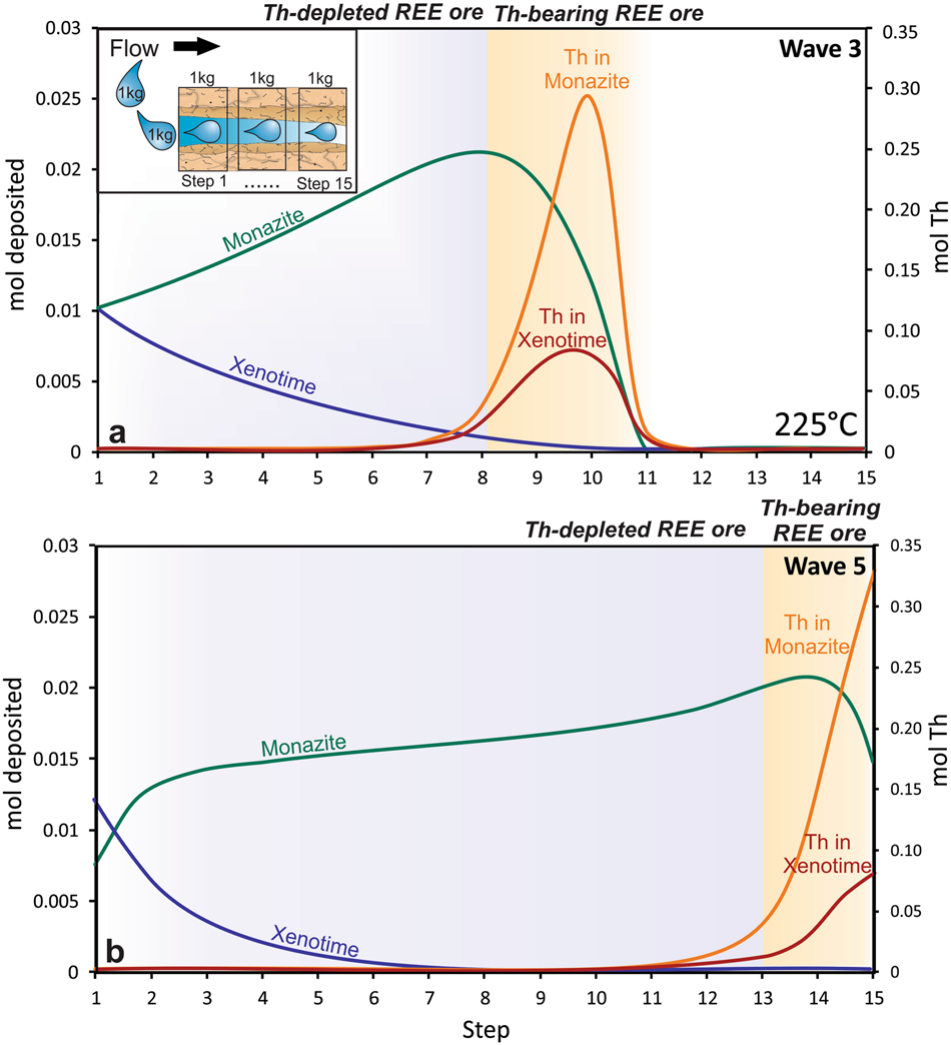
Results of a flow-through simulation of hydrothermal alteration. A step-flow reactor model was employed to investigate the fractionation of Th and the REE. The distribution of the REE-minerals, monazite and xenotime, and their Th contents at (**a**) wave 3 and (**b**) wave 5 in a column of apatite-bearing rock at 225 °C is shown. From this figure, it is evident that Th is concentrated at the alteration wave front due to its high mobility. After successive waves of fluid, Th is transported beyond the column, leaving behind REE-minerals depleted in Th.

To identify conditions in nature, which can lead to the separation of Th from the REE, we simulated the one-directional hydrothermal alteration of a column of rock containing 0.5 wt.% apatite-OH (Ca-hydroxy-phosphate, to allow for the formation of REE phosphates) by a REE and Th-bearing solution. To avoid alteration of the observed trends by pH and other buffering parameters, the rock was assumed to be chemically inert and the only chemically active component was apatite, which supplied the P needed to form two primary REE ore minerals, monazite (LREEPO_4_) and xenotime (HREEPO_4_). The association of these minerals in natural systems have been described previously^23,24^. The solution altering the rock contained 10 wt.% NaCl, 2 wt.% SO_4_^2−^, and had a pH of 2, consistent with the composition of some natural hydrothermal REE ore systems^20,25^. The simulation uses a step-flow reactor approach (“box model”; Fig. 3), which involves the interaction and initial equilibration of a 1 kg aliquot of solution with 1 kg of rock (“Step 1”). Monazite and xenotime were deposited as a result of the initial interaction. The equilibrated fluid then moved to the next reactor where it equilibrated with 1 kg of unaltered rock. This was repeated 15 times, creating a column of rock altered by an initial passage of the first fluid aliquot (“Wave 1”). After completion of this first wave, a new aliquot of fluid (1 kg) of identical composition was flushed through the newly altered column of rock in an identical manner (“Wave 2”). This process was repeated for a total of 10 waves, allowing us to predict how Th and the REE are likely to behave in well-evolved hydrothermal systems that experience continuous flushing of solution. For a complete description of the model, readers are referred to the Supplementary Information files.

At the temperature considered (225 °C), the simulation shows that Th is highly mobile in the modeled solution owing to the high stability of the Th(SO_4_)_2_ complex. Indeed, whereas Th-bearing monazite and xenotime solid solutions precipitated initially, subsequent flushing of the sulfate-bearing fluid through a REE deposit promoted the mobilization and concentration of Th in the wave front, resulting in the nearly complete removal of Th from the ore and the crystallization of Th-depleted monazite and xenotime behind the alteration front (Fig. 3). Our simulation provides two important guidelines for an exploration strategy for Th-depleted REE ore deposits. The first of these guidelines is that in choosing among potentially REE-rich targets, preference should be given to targets for which there is evidence of the passage of sulfate-rich fluids (e.g., the presence of sulfate minerals such as barite, anhydrite and gypsum, or from fluid inclusion studies). The second is that there should be evidence of intense, pervasive alteration by fluids having the composition required for the mobilization of Th from the ore. In contrast, if the object of exploration is the discovery of a Th deposit that can be used as a raw material for nuclear fuel, the alteration front, enriched with Th (Fig. 3), should be in close proximity to the zones depleted with Th; the latter could provide important vectors to economically exploitable Th mineralization.

The mechanism proposed in this model is dependent on the presence of SO_4_^2−^ as a ligand in the solution. The potential reduction of SO_4_^2−^ in natural systems can lead to the destabilization of Th-SO_4_ complexes and promote the deposition of Th-bearing minerals. It is known, however, that the thermal reduction of sulfate (TSR) is kinetically inhibited up to 300 °C^26^ and therefore the reduction of SO_4_^2−^ will likely not occur in many natural systems. Furthermore, it is worth noting that a potential restriction on the sulfate-mediated transport of Th is the retrograde solubility of most sulfate solids with temperature. In the calculations reported in this study, however, the concentrations of sulfate considered were taken directly from fluid inclusions of a natural REE hydrothermal system^27^. A related consideration is the reported occurrence of liquid-liquid immiscibility observed in multiple sulfate-bearing systems (e.g. UO_2_, Li, Mg^28–30^) at elevated temperatures (>285.8 °C, >336.5 °C, and >259.5 °C, respectively)^31^, which may serve to provide a source of excess sulfate for complexation. In these studies, aqueous sulfate solutions were observed to separate into a high density, sulfate-rich (up to 70% of total sulfate) phase and a low-density sulfate-depleted aqueous phase. Whether such liquid-liquid immiscibility can occur in nature or in nuclear industry applications and impact on the transport of Th in high temperature fluids is an intriguing question worthy of study.

This paper reports the first investigation of the behavior of Th at temperatures representative of hydrothermal systems. Contrary to the widely held view that Th is an immobile element, our results demonstrate that in high temperature sulfate-bearing solutions, ThO_2_ is extremely soluble, and therefore that Th is highly mobile. This is an important discovery in the field of actinide science that advances our understanding of the behavior of Th. Moreover, it is one that could promote the successful exploration for new, economically viable sources of the REE, facilitate the removal of Th from REE minerals, and potentially lead to new approaches in the fabrication and development of Th-based fuels.

## Materials and Methods

### Experimental procedure

The experiments involved investigating the solubility of Th dioxide (ThO_2_) in solutions as a function of varying sulfate concentration (0.05–0.5 m Na_2_SO_4_) at elevated temperatures (175, 200, 225, 250 °C) and the pressure of saturated water vapor. The experimental solutions were contained in Teflon reactors (50 mL PFA test tubes), which were placed inside Ti autoclaves treated with nitric acid to ensure their inertness (by creating a dense layer of TiO_2_ on the internal surfaces). Prior to each experiment, ThO_2_ powder (IBI Labs, Technical Grade 99.8%) was placed inside small quartz tubes capped with silica wool, and was exposed to conditions promoting hydrothermal re-crystallization until the grain sizes reached 10–50 μm. Thereafter, the tubes were placed in the Teflon reactors and submerged in their respective solutions (10–12 mL). The autoclaves were then sealed with a Teflon O-ring, and placed in a Muffle Furnace inside an Al box (with 1.5 cm thick walls to reduce temperature gradients) for 1–2 weeks. After heating, the autoclaves were removed from the furnace and immediately quenched in a stream of cold air (solutions reached ambient conditions in <25 min). The quartz tubes containing ThO_2_ were removed from the Teflon reactors, and the pH of the solutions was measured using a calibrated glass electrode. The electrode was calibrated with a set of NaCl/Na_2_SO_4_/HCl solutions having concentrations identical to those used in our experiments. Ion Chromatography (IC) was used to verify the sulfate content in solution. Subsequently, 2 mL of sulfuric acid (Fisher Scientific, TraceMetal Grade) was added to the experimental solutions and left for 24 hours for the purpose of dissolving any Th that may have precipitated on the walls of the Teflon reactors during quenching. Finally, the concentrations of Th in each solution were measured via Inductively Coupled Plasma Mass Spectrometry (ICP-MS) at the Geochemical and Geomaterials Research Laboratories of Los Alamos National Laboratory. A sketch of the experimental set up is shown in Supplementary Fig. S1.

The experimental solutions were prepared with a constant 1 molal NaCl concentration and Na_2_SO_4_ concentration varying from 0.05 to 0.5 molal to determine the solubility of Th as a function of sulfate activity. The addition of NaCl (Fisher Scientific, A.C.S. Grade) was necessary to satisfy the activity model used in our calculations, information for which can be found in the Supplementary Information file. The solutions were prepared with deionized water acidified with HCl (Fisher Scientific, TraceMetal Grade) to a pH_25 °C_ range of 2.01–2.67. Predetermined amounts of Na_2_SO_4_ (Fisher Scientific, A.C.S. Grade) were added to the solutions to set the activity of sulfate. Experimental parameters for each solution, including the calculated pH_T_ and activity of sulfate at the experimental temperatures, are reported in Supplementary Table S1.

The time required to attain equilibrium was determined in our previous study^15^, for NaCl-bearing solutions, to be less than 1 day at 175 °C, and thus we infer that for each of our experiments, which lasted >6 days at T ≥ 175 °C, the concentration of Th achieved an equilibrium/steady state. Phase characterization of the reference solid (ThO_2_) was performed by X-ray diffraction (XRD) to ensure that there was no phase change during the experiments (Supplementary Fig. S2).

## Supplementary information

Supplementary Information

Supplementary Dataset 1

## Data Availability

All data generated or analyzed during this study are included in this article (and its Supplementary Information files).
