# Supplementary material for: Challenging the thorium-immobility paradigm
Source: Sci Rep. 2019 Nov 19;9:17035. doi: 10.1038/s41598-019-53571-x (PMC6863889; doi:10.1038/s41598-019-53571-x)
Supplement: Supplementary file 1 — Supplementary Information [file 41598_2019_53571_MOESM1_ESM.pdf]

Supplementary Information for:

Challenging the thorium-immobility paradigm

Haylea Nisbet[1,2*], Artas Migdisov[1], Anthony Williams-Jones[2], Hongwu Xu[1], Vincent van Hinsberg[2], Robert Roback[1]

[1]Earth and Environmental Sciences Division, Los Alamos National Laboratory, Los Alamos, NM 87545, USA.
[2] Department of Earth and Planetary Sciences, McGill University, 3450 University Street, Montreal, QC, H3A 0E8, Canada.

Supplementary Information Text

Results and data treatment

Phase characterization of the reference solid (ThO_2) was performed by X-ray Diffraction (XRD) in order to ensure no phase change occurred (i.e., $Th(SO_4)_{2(solid)}$) during the experiments. An example of the XRD spectra obtained from solids taken at the end of multiple experiments is illustrated in Supplementary Fig. S2, and shows that, indeed, the solid remained ThO_2 and that there are no peaks for $Th(SO_4)_2$.

The results of the solubility experiments are presented in Supplementary Table S1, which lists the experimental parameters for each solution, the logarithm of the molality of Th measured, the activity of sulfate calculated at the experimental conditions, the pH measured after completion of the experiments ($pH_{25°C}$), and the pH extrapolated to the experimental temperature (pH_T). The pH at the experimental temperature (pH_T) differs from the pH measured at ambient conditions ($pH_{25°C}$) owing to changes in the dissociation constant of water and the dissolved species. In order to determine the pH_T, the thermodynamic modeling software HCh was used[1]. The thermodynamic model employed for these calculations involved the following species: H_2O, H^+, OH^-, O_2, H_2, Na^+, NaOH°, $NaSO_4^-$, NaCl°, SO_4^{2-}, HSO_4^-, Cl^-, and HCl° and thermodynamic data for modeling the aqueous solutions at each experimental temperature were taken from Refs. [2–4]. The dissociation constant and thermodynamic properties of water were calculated using the Marshall and Frank model [5] and the Haar-Gallagher-Kell model [6], respectively. Initially, the composition of the experimental solution was modeled to determine the concentration of HCl at 25°C corresponding to the experimentally measured $pH_{25°C}$. Subsequently, the pH was recalculated at the experimental temperature (pH_T) using the measured concentrations of HCl. The activity model used in these and all subsequent calculations (see below) was the Debye-Hückel model modified by Refs. [7–9], recommended for NaCl-dominated solutions up to I=6 and temperatures up to 600°C:

$$\log \gamma_i = -\frac{AZ_i^2\sqrt{I}}{1+B\dot{a}\sqrt{I}} + b_\gamma I + \Gamma \qquad \text{(S1)}$$

where A and B are the Debye-Hückel solvent parameters, γ_i, Z_i and $\dot{a}_i$ are the individual molal activity coefficient, the charge, and the distance of closest approach of an ion i,

respectively. The effective ionic strength calculated using the molal scale is I, Γ is a molarity to molality conversion factor, and b_γ is the extended-term parameter for NaCl from Refs. [8] and [9].

Calculation of equilibrium constants

As reported in Supplementary Table S1, the pH of the experimental solutions varied within 0.5 log units for the range of conditions of our experimental solutions. In order to identify the Th-sulfate species from the stoichiometric slope of our experimental data, we normalized the pH based on the two species that have been observed in sulfate-bearing solutions at acidic to moderately acidic conditions ($ThSO_4^{2+}$ and $Th(SO_4)_2$):

$$ThO_2(s) + 2SO_4^{2-} + 4H^+ \leftrightarrow Th(SO_4)_2 + 2H_2O \tag{S2}$$

$$ThO_2(s) + SO_4^{2-} + 4H^+ \leftrightarrow Th(SO_4)^{2+} + 2H_2O \tag{S3}$$

As shown in Fig. 1, which plots the normalized values for the logarithm of the concentration of Th as a function of the logarithm of the activity of sulfate, for each isotherm, the concentration of Th increases with increasing activity of sulfate with a slope of approximately 2. Based on the stoichiometric slope, the results suggest that the aqueous species controlling the solubility of ThO_2 in our experiments is most likely $Th(SO_4)_2$, formed through Reaction S2.

The activity of sulfate was calculated using the same model as that employed for the pH_T calculations. The activity values, which are listed in Supplementary Table S1, differ significantly from the total sulfate molality in the solutions. This difference is primarily due to the range of pH_T of the experimental solutions, which is primarily in the predominance field of HSO_4^-, but extends into the sulfate field at lower temperature (Supplementary Fig. S3). Thus, the concentrations of sulfate in most cases represent a minor proportion of the total concentration of sulfate. In addition, because sulfate is a charged species, it is strongly influenced by the ionic strength of the solutions.

The experimental data reported in Supplementary Table S1 were used to calculate equilibrium constants for Reaction S2 for each isotherm investigated. The values of the derived constants are reported in Table 1. The calculations accounted for the following species: Th^{4+}, ThO_2, $Th(OH)_2^{2+}$, $Th(OH)_4$, and $Th(SO_4)_2$. Data for Th^{4+} and thorianite

(ThO_2) were taken from Refs. [10] and [11], respectively. Data for Th hydroxyl species are taken from Ref. [12]. The contribution of polynuclear species to the solubility of Th was not considered as it can be assumed that these species become unstable at high temperature due to the large increase of electrostatic repulsion associated with the decrease of the dielectric constant of water [13], as demonstrated in Ref. [12].

Extrapolation to low temperature and comparison to previous studies

To the best of our knowledge, this is the first study to investigate Th-sulfate speciation at hydrothermal conditions. The other thermodynamic data for $Th(SO_4)_2$ are restricted to ambient conditions, and are reported in an extensive review performed by the Nuclear Energy Agency (NEA) [14]. This report cites several studies that have derived equilibrium constants at ambient conditions for $Th(SO_4)_2$ and $ThSO_4^{2+}$; the species expected to predominate in an aqueous solution at low temperature [15–18]. These studies invoked liquid-liquid extraction methods with thenoyltrifluoroacetone (TTA) or dinonyl naphthalene sulphonic acid (DNNS) as extracting ligands, and by using ion-exchange. The experiments involved fluids with relatively high acidity and a near constant ionic strength of 1.7-2.0 M [14]. Variation among the derived equilibrium constants for the $Th(SO_4)_2$ complex from these studies is small. However, in order to accurately compare these values, they were recalculated to zero ionic strength by NEA using a modified NONLINT-SIT code [19]. These values were then averaged and combined with the equilibrium constant for the protonation of sulfate to obtain a thermodynamic formation constant of $\log \beta_2 = 9.69 \pm 0.27$. Recently, Ref. [20] reported results of calorimetric titration experiments for temperatures between 10-70°C and derived equilibrium constants and enthalpies of complexation for the Th-sulfate complexes. The reported formation constants for $Th(SO_4)_2$ at 10, 25, 40, 55, and 70°C were 9.48 ± 0.09, 9.99 ± 0.10, 10.45 ± 0.10, 10.56 ± 0.10, and 11.10 ± 0.10, respectively. In order to compare our data with those reported in previous studies, the formation constants obtained from this study were extrapolated to low temperature by fitting the $\log \beta_2$ for each temperature to the Ryzhenko-Bryzgalin model (MRB) [21] modified by Ref. [22] as described in Ref. [23]. This model fits the temperature and pressure dependence of the dissociation constant for ion pairs through the following equation:

$$logK_{(T,P)} = \frac{T_r}{T} logK_{(T_r P_r)} + B_{(T,P)} \left(A_{zz/a} + \frac{B_{zz/a}}{T} \right) \quad \text{(S4)}$$

where K is the dissociation constant of the ion pair, T_r and P_r are the reference temperature and pressure, $B_{(T,P)}$ accounts for the property of water at the temperature and pressure calculated from data contained in Ref. [5], and $A_{zz/a}$ and $B_{zz/a}$ are the fitting parameters. The parameters derived from this model for $Th(SO_4)_2$ are found in Supplementary Table S2. Supplementary Fig. S4 shows the thermodynamic formation constants from this study extrapolated to 10°C for comparison with the selected constant from the NEA review [14], and values derived from Ref. [20]. As shown in this figure, the formation constants calculated in this study systematically increase with temperature, and when back-extrapolated to low temperature, show excellent agreement with the previously reported values.

Modeling

The model presented in this contribution simulates progressive hydrothermal alteration and re-distribution of REE and Th by an acidic solution in a one-dimensional column of a rock containing 0.5 wt. % apatite-OH (Ca-hydroxy-phosphate, to allow for the formation of REE phosphates), which was evaluated using a step-flow reactor approach, similar to Refs. [24] and [25] (‘‘box model”, Fig. 3). The calculations were performed for 225°C to be within the range of experimental data for Th. To avoid changes in the observed trends by pH, and other buffering parameters, the rock was assumed to be chemically inert and the only chemically active component of it was apatite, which supplied the P needed to form monazite or xenotime. The composition of the altering solution corresponded to compositions documented for fluids associated with natural REE ore forming systems. Fluid inclusion studies indicate that the Bayan Obo deposit in China was formed from brines containing 7–10 wt.% NaCl equivalent [26], whereas fluids responsible for ore deposition at Gallinas Mountains contained 12–18 wt.% NaCl equivalent [27]. To avoid uncertainties associated with poorly defined activity models for highly saline brines at elevated temperature, the solution selected for the simulations contained only 10 wt.% NaCl (1.72 mol/kg). The initial concentrations of REE in the solution associated with the initial depositional event (‘‘step 1”) were closely approximated to the of fluid inclusions from the Capitan Pluton REE prospect [28] (Supplementary Table S3). This publication, however, did not report the concentration of Y, the main component of xenotime solid

solutions. In our simulations, the concentration of this element was set to be one third of the concentrations of La and Ce. The concentration of Th in the solution was fixed by its saturation with respect to thorianite (ThO_2) at 225 °C. The pH_T of the initial solution was set at ~2 by adding the required amount of HCl.

A full list of aqueous species employed in the calculations, the parameters for the equations of state used to extrapolate their properties to elevated temperature, and their data sources, is provided in the Supplementary Dataset. Calculations of the thermodynamic properties of the species and the H_2O dissociation constant were performed using the same models as those mentioned above. Similarly, the activity of the individual ions was calculated using the modified extended Debye-Hückel model (Eqn. S1). The thermodynamic properties of basic aqueous species $O^o_{2\,aq}$, $H^o_{2\,aq}$, Na^+, $NaOH^o_{aq}$, $NaSO_4^-$, $NaCl^o_{aq}$, and Cl^- were taken from Refs. [2,3] and [10]. The stability of the HCl^o_{aq} ion pair was evaluated using the combined data from Refs. [4] and [29]. The properties of simple hydrated ions REE^{3+} and Th^{4+} were taken from Ref. [10]. The thermodynamic data for REE and Th aqueous species incorporated in this model are identical to those described in Ref. [25]. Exceptions are the REE-sulfate complexes, $REESO_4^+$ and $REE(SO_4)_2^-$ [30] and the data derived in this contribution for $Th(SO_4)_2$. For more detail on the sources and selection of thermodynamic data, readers are referred to Refs. [25] and [30].

The model mainly investigates the incorporation of Th in REE phosphate solid solutions and co-existing aqueous phases. Additionally, it incorporates co-existing thorianite (ThO_2 [11]), solid REE-hydroxides [30], apatite-OH, whitlockite ($Ca_5(PO_4)_2$), halite (NaCl), hydrophilite ($CaCl_2$), and portlandite ($Ca(OH)_2$)[11]. Except for thorianite, apatite-OH, and whitlockite, all phases mentioned above were unstable under the conditions employed in our model. Thermodynamic properties for monazite end-members ($LaPO_4$ to $GdPO_4$) were derived from calorimetric measurements reported by Refs. [31–33] and solubility products determined for REE phosphates at 25 °C [34]. Thermodynamic properties for xenotime were derived from calorimetric data reported in Refs. [33,35–38] and the solubility products at 25°C reported in Ref. [34]. For details on data selection, readers are referred to Ref. [25].

To determine the mixing systematics of L/MREE in monazite and HREE in xenotime, a regular solid solution model was employed in which the excess enthalpy of mixing ΔH_{mix}^{E} is expressed as $W \cdot x(1-x)$, where W is the interaction parameter and x is the portion of one REE over the cation site. The interaction parameters for the monazite system were derived based on *ab initio* calculations from Refs. [39,40], where the excess mixing properties of two REE in monazite were expressed as a function of volume mismatch from the two end-members, proportional to the average Young's modulus $\bar{E}$. The derivation of the xenotime system interaction parameters first involved the calculation of E values, which were estimated based on their near linear correlation with the ionic radii of the cations [40]. These values were then used to calculate W for each substituting pair of HREE. The incorporation of Th into monazite and xenotime was modeled through the brabantitic substitution, in which the extra charge, introduced by the tetravalent actinide, is accommodated via incorporation of Ca^{2+} into the structure. Thermodynamic properties of the pure $(Ca,Th)PO_4$ end-member were taken from Refs. [41] and [42]. For a detailed description of the solid solution model, readers are referred to Ref. [25].

The most common varieties of monazite in nature are monazite-(Ce) and monazite-(La). Monazite-(Nd) is also found in natural systems but is much rarer. Thus, in our model we accounted for two types of monazite: monazite-(Ce), containing $CePO_4$, $PrPO_4$, $NdPO_4$, $SmPO_4$, $GdPO_4$, and $(Ca,Th)PO_4$, and monazite-(La) containing $LaPO_4$, $PrPO_4$, $NdPO_4$, $SmPO_4$, $GdPO_4$, and $(Ca,Th)PO_4$. This separation into two types of monazite is necessary, due in part to limitations of the software (HCh) [23], which cannot account for solid solutions having more than seven components. Formation of monazite-(Nd) was assumed when $NdPO_4$ predominated in one of the above solid solutions. The xenotime solid solution contained YPO_4, $TbPO_4$, $DyPO_4$, $ErPO_4$, $YbPO_4$, and $(Ca,Th)PO_4$.

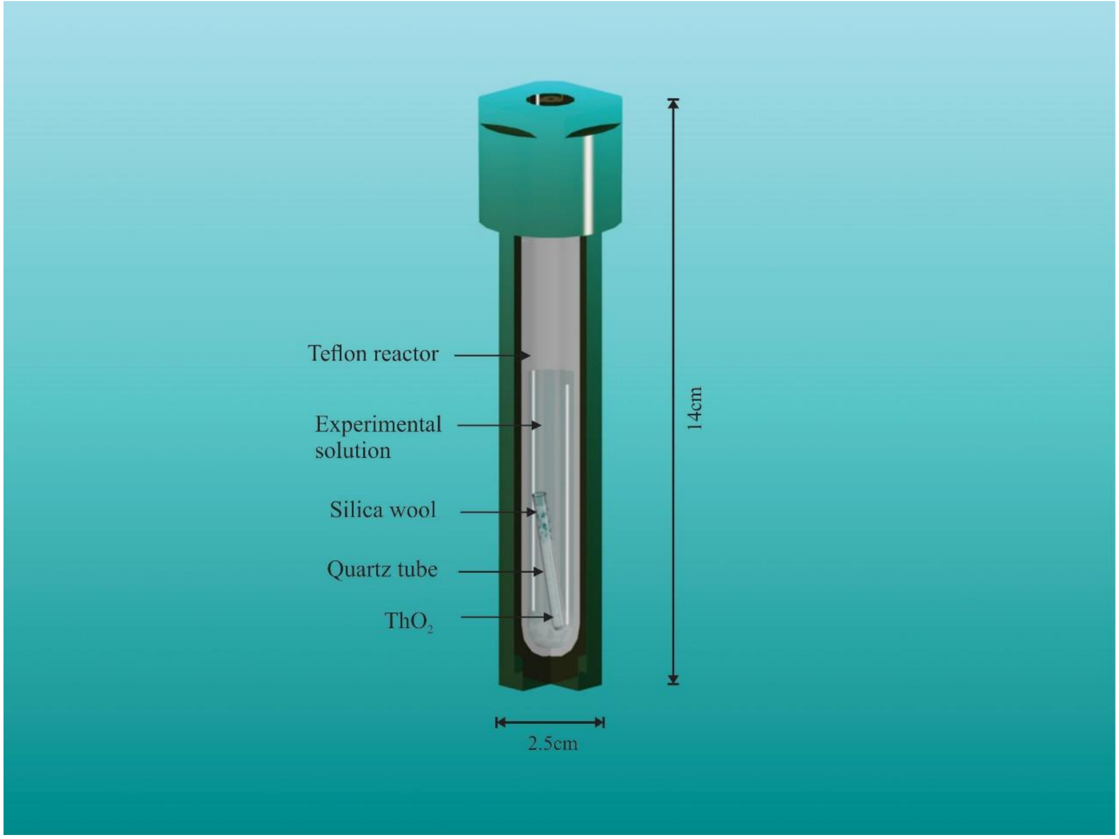

Supplementary Figure S1: A sketch of the experimental set up. Experimental solutions were contained in titanium autoclaves lined with Teflon. ThO_2 was placed inside a small quartz tube capped with silica wool. Approximately 10ml of experimental solution was added.

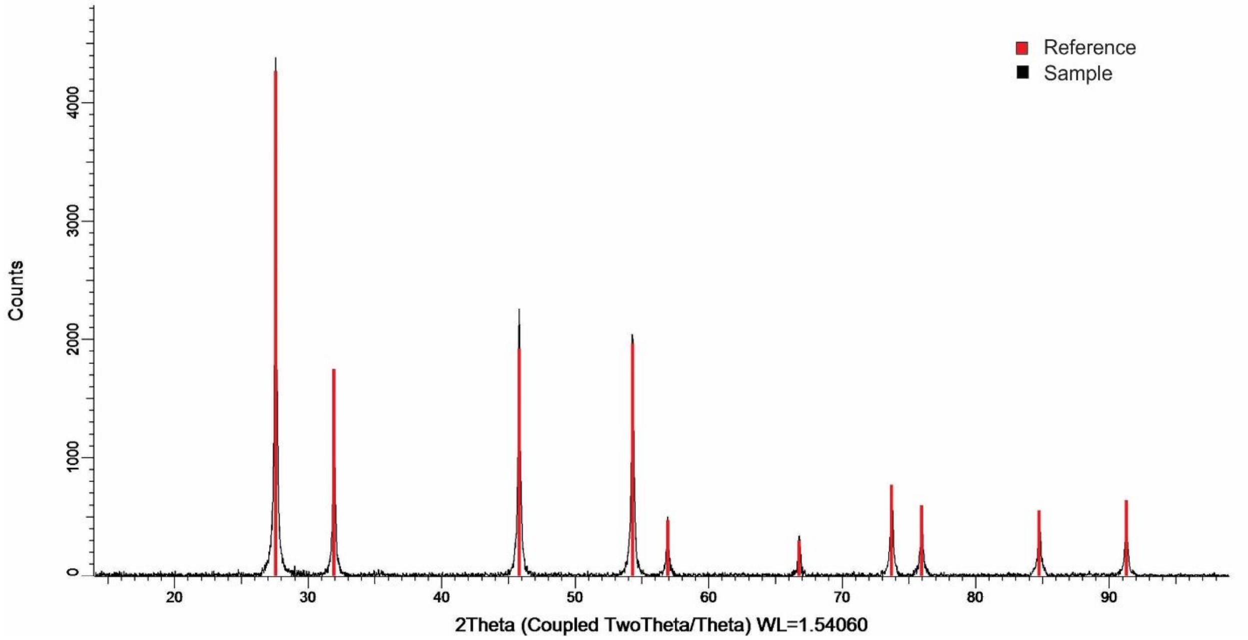

Supplementary Figure S2: X-Ray Diffractogram (XRD) for ThO_2 after completion of experiments. The ThO_2 was analyzed at the end of multiple experiments and analyzed by XRD to verify the phase remained the same. As illustrated, the solid is ThO_2.

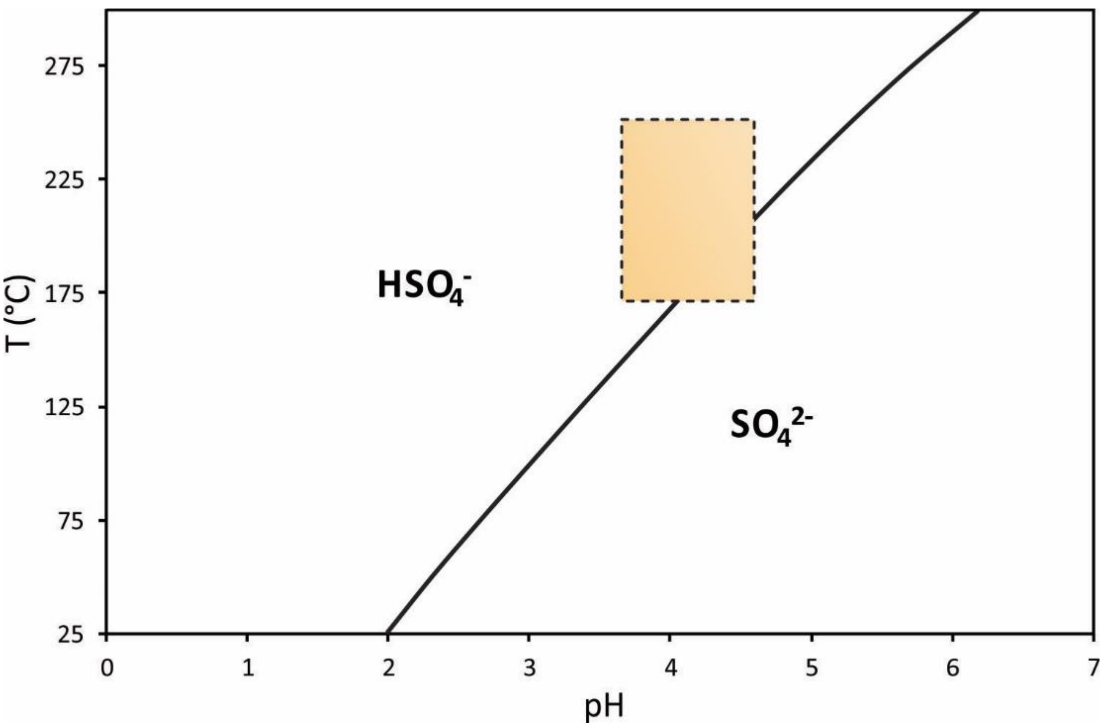

Supplementary Figure S3: Sulfate predominance diagram with respect to temperature and pH. The orange box corresponds to the field of our experimental data, showing that both sulfate species were present under the experimental conditions.

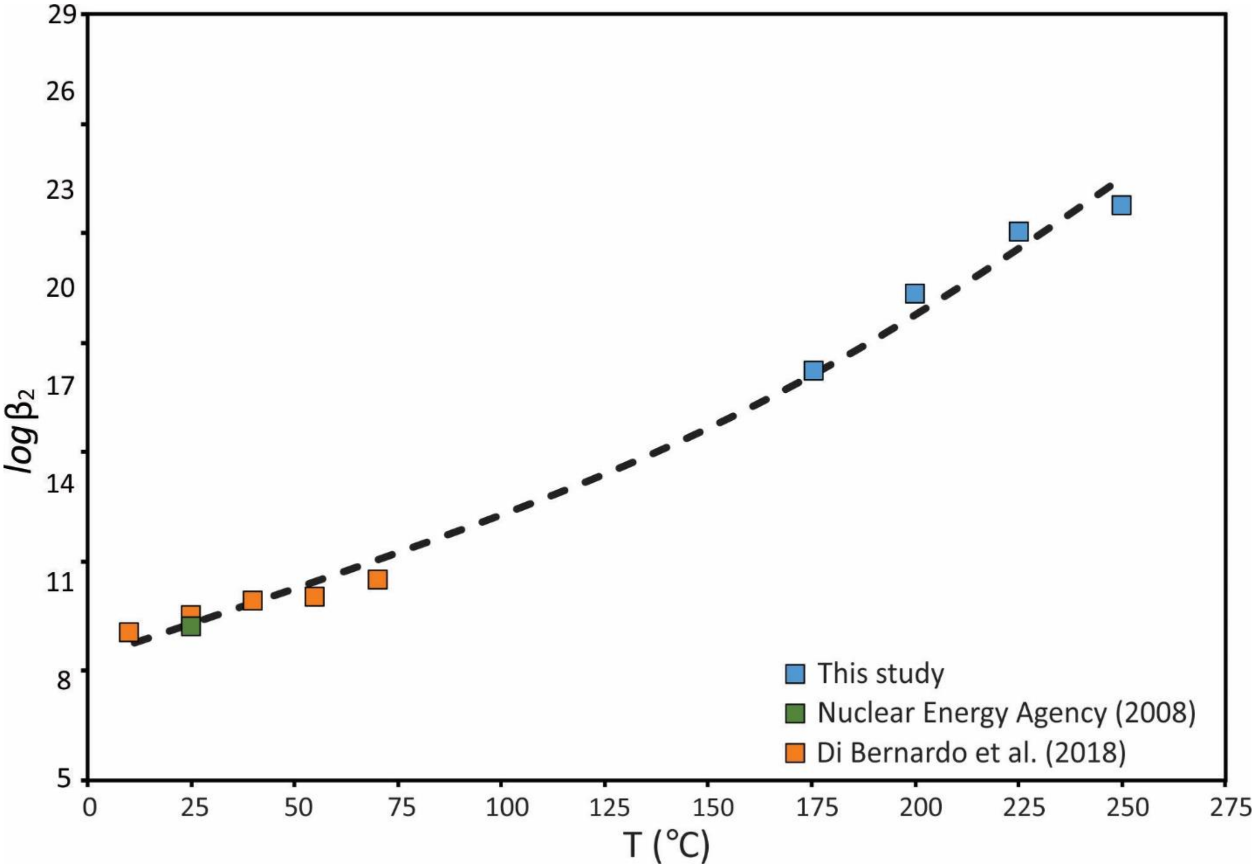

Supplementary Figure S4: Extrapolation and comparison of calculated thermodynamic formation constants from this study to low temperature. Formation constants ($\log \beta_2$) for $Th(SO_4)_2$ plotted as a function of temperature. The experimental data obtained in this study (blue squares) were fitted to the Bryzgalin–Ryzhenko model (dashed line) to compare with the selected data from the Nuclear Energy Agency [14] (green square) and data collected from Ref. [20] (orange squares). The error (SD) associated with the individual points is smaller than the size of the markers. Our data shows excellent agreement with the low temperature values.

Supplementary Table S1: The composition of the experimental solutions. Concentrations of the components in the experimental solutions and pH values measured after quenching (25°C) and extrapolated to the temperature of the experiments (pH_T).

T (°C)	log m Na_2SO_4	log a SO_4^{2-}	$pH_{25°C}$	pH_T	log m Th	log m Th (pH corrected)
175	-1.301	-3.030	2.44	3.69	-8.466	-8.466
175	-1.0	-2.739	2.66	3.88	-8.467	-8.096
175	-0.824	-2.586	2.55	3.85	-8.247	-7.935
175	-0.456	-2.289	2.47	3.89	-7.012	-6.608
175	-0.347	-2.204	2.63	4.08	-7.604	-6.838
175	-0.301	-2.172	2.63	4.09	-7.834	-7.046
200	-1.301	-3.181	2.71	3.99	-7.766	-7.766
200	-1.0	-2.895	2.67	4.13	-8.026	-7.750
200	-0.824	-2.740	2.55	4.16	-7.845	-7.509
200	-0.699	-2.632	2.63	4.25	-7.971	-7.461
200	-0.602	-2.557	2.60	4.20	-7.703	-7.287
200	-0.523	-2.493	2.67	4.30	-7.805	-7.185
200	-0.456	-2.443	2.63	4.31	-7.652	-7.026
200	-0.347	-2.365	2.67	4.38	-7.267	-6.495
225	-1.0	-3.079	2.56	4.26	-7.762	-7.762
225	-0.824	-2.922	2.54	4.32	-7.432	-7.300
225	-0.699	-2.815	2.56	4.40	-7.621	-7.345
225	-0.602	-2.737	2.57	4.44	-7.445	-7.073
225	-0.347	-2.554	2.54	4.48	-7.458	-7.010
225	-0.301	-2.522	2.58	4.54	-7.283	-6.721
250	-1.301	-3.595	2.44	4.16	-8.567	-8.567
250	-1.0	-3.356	2.09	3.96	-7.525	-7.922
250	-0.824	-3.180	2.09	4.08	-8.058	-8.222
250	-0.699	-3.041	2.29	4.36	-8.170	-7.770
250	-0.602	-2.947	2.49	4.61	-8.106	-7.198
250	-0.523	-2.899	2.32	4.46	-7.667	-7.071
250	-0.347	-2.785	2.20	4.38	-7.198	-6.758
250	-0.347	-2.773	2.36	4.55	-7.824	-7.044

Supplementary Table S2: Ryzhekno-Bryzgalin model (MRB) parameters. Thermodynamic formation constants derived in this study for $Th(SO_4)_2$ fitted to the Ryzhekno-Bryzgalin Model and model parameters.

	log β				pK(298)	A(zz/a)	B(zz/a)
	175°C	200°C	225°C	250°C			
$Th(SO_4)_2$	17.93	19.51	21.16	22.93	9.763	7.231	-877.53

Supplementary Table S3: Concentrations of REE in the initial modeling solution. Initial concentrations of the REE in the solution associated with the initial depositional event (‘‘step 1”). The values are close to those of the Capitan Pluton REE fluid [28].

Element	La	Ce	Pr	Nd	Sm	Gd	Y	Tb	Dy	Er	Yb
Concentration (ppm)	300	300	35	150	20	20	100	6	45	33	33

Supplementary References

1. Shvarov, Y. V. OptimA: A program for the calculation of the free energies of dissolved aqueous species from the results of chemical experiments. (2010).
2. Johnson, J. W., Oelkers, E. H. & Helgeson, H. C. SUPCRT92: a software package for calculating the standard molal thermodynamic properties of minerals, gases, aqueous species, and reactions from 1 to 5000 bar and 0 to 1000 °C. *Comput. Geosci.* **18**, 899–947 (1992).
3. Sverjensky, D. A., Shock, E. L. & Helgeson, H. C. Prediction of the thermodynamic properties of aqueous metal complexes to 1000 °C and 5 kb. *Geochim. Cosmochim. Acta* **61**, 1359–1412 (1997).
4. Tagirov, B. R., Zotov, A. & Akinfiev, N. Experimental study of dissociation of HCl from 350 to 500 °C and from 500 to 2500 bars: thermodynamic properties of HCl°(aq). *Geochim. Cosmochim. Acta* **61**, 4267–4280 (1997).
5. Marshall, W. L. & Franck, E. U. Ion product of water substance, 0-1000 °C, 1-10,000 bars new International Formulation and its background. *J. Phys. Chem. Ref. Data* **10**, 295–304 (1981).
6. Kestin, J., Sengers, J. V., Kamgar-Parsi, B. & Levelt Sengers, J. M. H. Thermophysical properties of fluid H2O. *J. Phys. Chem. Ref. Data* **13**, 601–609 (1984).
7. Helgeson, H. C., Kirkham, D. H. & Flowers, G. C. Theoretical prediction of the thermodynamic behavior of aqueous electrolytes at high pressures and temperatures: IV. Calculation of activity coefficients, osmotic coefficients, and apparent molal and standard and relative partial molal properties to 600°C. *Am. J. Sci.* **281**, 1249–1516 (1981).
8. Oelkers, E. H. & Helgeson, H. C. Calculation of activity coefficients and degrees of formation of neutral ion pairs in supercritical electrolyte solutions. *Geochim. Cosmochim. Acta* **55**, 1235–1251 (1991).
9. Oelkers, E. H. & Helgeson, H. C. Triple-ion anions and polynuclear complexing in supercritical electrolyte solutions. *Geochim. Cosmochim. Acta* **54**, 727–738 (1990).

10. Shock, E. L., Sassani, D. C., Willis, M. & Sverjensky, D. A. Inorganic species in geological fluids: Correlations among standard molal thermodynamic properties of aqueous ions and hydroxide complexes. *Geochim. Cosmochim. Acta* **61**, 907–950 (1997).

11. Robie, R. A. & Hemingway, B. S. Thermodynamic properties of minerals and related substances at 298.15 K and 1 Bar (10^5 Pascals) pressure and at higher temperatures. *US Geol. Survet Bull.* **2131**, 461 (1995).

12. Nisbet, H. *et al.* An experimental study of the solubility and speciation of thorium in chloride-bearing aqueous solutions at temperatures up to 250 °C. *Geochim. Cosmochim. Acta* **239**, 363–373 (2018).

13. Seward, T. M., Williams-Jones, A. E. & Migdissov, A. A. The Chemistry of Metal Transport and Deposition by Ore-Forming Hydrothermal Fluids. *Treatise Geochem.* **10**, 29–57 (2013).

14. Rand, M. H., Mompean, F. J., Perrone, J. & Illemassene, M. *Chemical Thermodynamics of Thorium*. (OECD, NEA, 2008).

15. Maiorova, E. P. & Fomin, V. V. Extraction of thorium with tributyl phosphate. III. Effect of sulfate ions on the distribution of thorium. *Russ. J. Inorg. Chem.* **3**, 295–316 (1958).

16. Zebroski, E. L., Alter, H. W. & Heumann, F. K. Thorium Complexes with Chloride, Fluoride, Nitrate, Phosphate and Sulfate. *J. Am. Chem. Soc.* **73**, 5646–5650 (1951).

17. Patil, S. K. & Ramakrishna, V. V. Study of the sulphate complexing of Th(IV) by solvent extraction with dinonyl naphthalene sulphonic acid. *Radiochim. Acta* **18**, 190–192 (1972).

18. Zielen, A. J. Thermodynamics of the sulfate complexes of thorium. *J. Am. Chem. Soc.* **81**, 5022–5028 (1959).

19. Felmy, A. R. A Computerized chemical equilibrium program using a constrained minimization of the Gibbs free energy: Summary report. *SSSA Spec. Publ. Soil Sci. Soc. Am. Am. Soc. Agron.* 377–407 (1995).

20. Di Bernardo, P. *et al.* Complexation of Th(IV) with sulfate in aqueous solution at 10–70 °C. *J. Chem. Thermodyn.* **116**, 273–278 (2018).

21. Ryzhenko, B. N., Bryzgalin, O. V., Artamkina, I. Y., Spasennykh, M. Y. & Shapkin, A. I. An electrostatic model for the electrolytic dissociation of inorganic substances dissolved in water. *Geochem. Int.* **22**, 138–144 (1985).

22. Borisov, M. V. & Shvarov, Y. V. Thermodynamics of geochemical processes. *Mosc. Mosc. State Univ. Publ. House* 254 (1992).

23. Shvarov, Y. V. & Bastrakov, E. HCh, A Software Package for Geochemical Equilibrium Modeling: User's Guide. (1999).

24. Gysi, A. P. & Williams-Jones, A. E. Hydrothermal mobilization of pegmatite-hosted REE and Zr at Strange Lake, Canada: A reaction path model. *Geochim. Cosmochim. Acta* **122**, 324–352 (2013).

25. Migdisov, A., Guo, X., Nisbet, H., Xu, H. & Williams-Jones, A. E. Fractionation of REE, U, and Th in natural ore-forming hydrothermal systems: Thermodynamic modeling. *J. Chem. Thermodyn.* **128**, 305–319 (2019).

26. Smith, M. P. & Henderson, P. Preliminary Fluid Inclusion Constraints on Fluid Evolution in the Bayan Obo Fe-REE-Nb Deposit, Inner Mongolia, China. *Econ. Geol.* **95**, 1371–1388 (2000).

27. Williams-Jones, A. E., Samson, I. M. & Olivo, G. R. The Genesis of Hydrothermal Fluorite-REE Deposits in the Gallinas Mountains, New Mexico. *Econ. Geol.* **95**, 327–342 (2000).

28. Banks, D. A., Yardley, B. W. D., Campbell, A. R. & Jarvis, K. E. REE composition of an aqueous magmatic fluid: A fluid inclusion study from the Capitan Pluton, New Mexico, U.S.A. *Chem. Geol.* **113**, 259–272 (1994).

29. Ho, P. C., Palmer, D. A. & Gruszkiewicz, M. S. Conductivity Measurements of Dilute Aqueous HCl Solutions to High Temperatures and Pressures Using a Flow-Through Cell. *J. Phys. Chem. B* **105**, 1260–1266 (2001).

30. Migdisov, A., Williams-Jones, A. E., Brugger, J. & Caporuscio, F. A. Hydrothermal transport, deposition, and fractionation of the REE: experimental data and thermodynamic calculations. *Chem. Geol.* **439**, 13–42 (2016).

31. Popa, K. & Konings, R. J. M. High-temperature heat capacities of EuPO4 and SmPO4 synthetic monazites. *Thermochim. Acta* **445**, 49–52 (2006).

32. Popa, K., Sedmidubský, D., Beneš, O., Thiriet, C. & Konings, R. J. M. The high-temperature heat capacity of LnPO4 (Ln=La, Ce, Gd) by drop calorimetry. *J. Chem. Thermodyn.* **38**, 825–829 (2006).

33. Navrotsky, A. *et al.* Thermodynamics of solid phases containing rare earth oxides. *J. Chem. Thermodyn.* **88**, 126–141 (2015).

34. Liu, X. & Byrne, R. H. Rare earth and yttrium phosphate solubilities in aqueous solution. *Geochim. Cosmochim. Acta* **61**, 1625–1633 (1997).

35. Gavrichev, K. S. *et al.* Thermodynamic functions of erbium orthophosphate ErPO4 in the temperature range of 0–1600K. *Thermochim. Acta* **535**, 1–7 (2012).

36. Gavrichev, K. S., Ryumin, M. A., Tyurin, A. V., Gurevich, V. M. & Komissarova, L. N. Heat capacity and thermodynamic functions of xenotime YPO4(c) at 0–1600 K. *Geochem. Int.* **48**, 932–939 (2010).

37. Gavrichev, K. S. *et al.* Heat capacity and thermodynamic functions of LuPO4 in the range 0–320K. *Thermochim. Acta* **448**, 63–65 (2006).

38. Gavrichev, K. S. *et al.* Heat capacity and thermodynamic functions of YbPO4 from 0 to 1800 K. *Inorg. Mater.* **49**, 701–708 (2013).

39. Kowalski, P. M. & Li, Y. Relationship between the thermodynamic excess properties of mixing and the elastic moduli in the monazite-type ceramics. *J. Eur. Ceram. Soc.* **36**, 2093–2096 (2016).

40. Mogilevsky, P. On the miscibility gap in monazite–xenotime systems. *Phys. Chem. Miner.* **34**, 201–214 (2007).

41. Popa, K. *et al*. Thermodynamic properties of CaTh(PO4)2 synthetic cheralite. *Am. Mineral.* **93**, 1356–1362 (2008).

42. Rawat, D., Phapale, S., Mishra, R. & Dash, S. Thermodynamic studies on charge-coupled substituted synthetic monazite. *J. Nucl. Mater*. **487**, 406–417 (2017).
